# Ag Vacancies as “Killer-Defects” in CaAgSb Thermoelectrics

**DOI:** 10.1021/acsaem.4c02907

**Published:** 2025-02-11

**Authors:** A. K. M. Ashiquzzaman Shawon, Ferdaushi Alam Bipasha, Channyung Lee, Kamil M. Ciesielski, Brian Tijan, Eric S. Toberer, Elif Ertekin, Alexandra Zevalkink

**Affiliations:** † Department of Chemical Engineering and Material Science, Michigan State University, East Lansing, Michigan 48824, United States; ‡ Department of Mechanical Science and Engineering, University of Illinois at Urbana−Champaign, Urbana, Illinois 61801, United States; § Department of Physics, Colorado School of Mines, Golden, Colorado 80401, United States

**Keywords:** thermoelectric, Zintl phase, phase-boundary mapping, defect calculations, carrier concentration

## Abstract

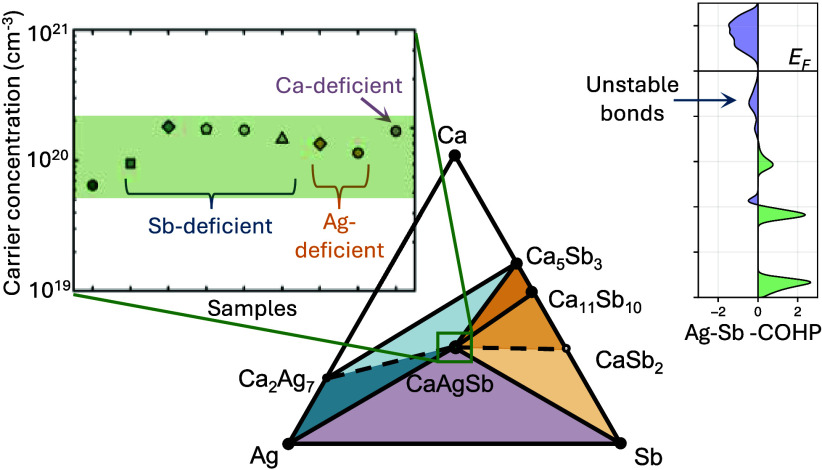

The *AMX* Zintl compound CaAgSb was recently identified as a promising thermoelectric material with high hole mobility and low lattice thermal conductivity. The single parabolic band model predicts that a *zT* of ∼1 can be achieved if the carrier concentration can be tuned to ∼10^19^ cm^–3^. However, the high inherent *p*-type carrier concentration of ∼10^20^ cm^–3^ in CaAgSb has limited further optimization of *zT* in *p*-type samples and has prevented *n*-type doping. In this work, we use a combination of computational and experimental tools to study the Fermi-level tunability of CaAgSb. Defect calculations based on density functional theory (DFT) reveal that acceptor-type defects, in particular Ag-vacancies, are the dominant defect across the full chemical potential space. This pins the Fermi energy within the valence band, leading to predicted *p*-type carrier concentrations that fluctuate within a narrow range. Crystal Orbital Hamilton Population (COHP) analysis shows that the Ag–Sb antibonding orbitals lie below the Fermi energy, which may explain the low Ag-vacancy formation energy in CaAgSb. Experimentally, we used a phase boundary mapping approach to explore the defect chemistry under different synthesis conditions. Samples were synthesized in the Ca-rich, Ag-rich, and Sb-rich regions of the phase diagram, and all were found to have high *p*-type carrier concentrations, ranging from 6.0 × 10^19^ to 1.8 × 10^20^ cm^–3^, and therefore similar thermal and electronic properties, consistent with the defect calculations. Taken together, our results confirm that Ag vacancies act as killer defects in CaAgSb, posing the primary challenge for further improvement of thermoelectric performance.

## I Introduction

Thermoelectric (TE) generators are solid-state devices that convert thermal energy to electrical energy with no moving parts.^1^ This makes them extremely useful for niche applications like deep-space probes, and *potentially* useful for broader applications in waste heat recovery.^2^ The power generation efficiency of TE devices is directly related to the materials parameter, , where *S* is the Seebeck coefficient, *ρ* is the resistivity, *κ* is the total thermal conductivity and *T* is the absolute temperature.^1,3^ These physical parameters are interdependent, which makes optimizing *zT* challenging.

**Figure 1:**
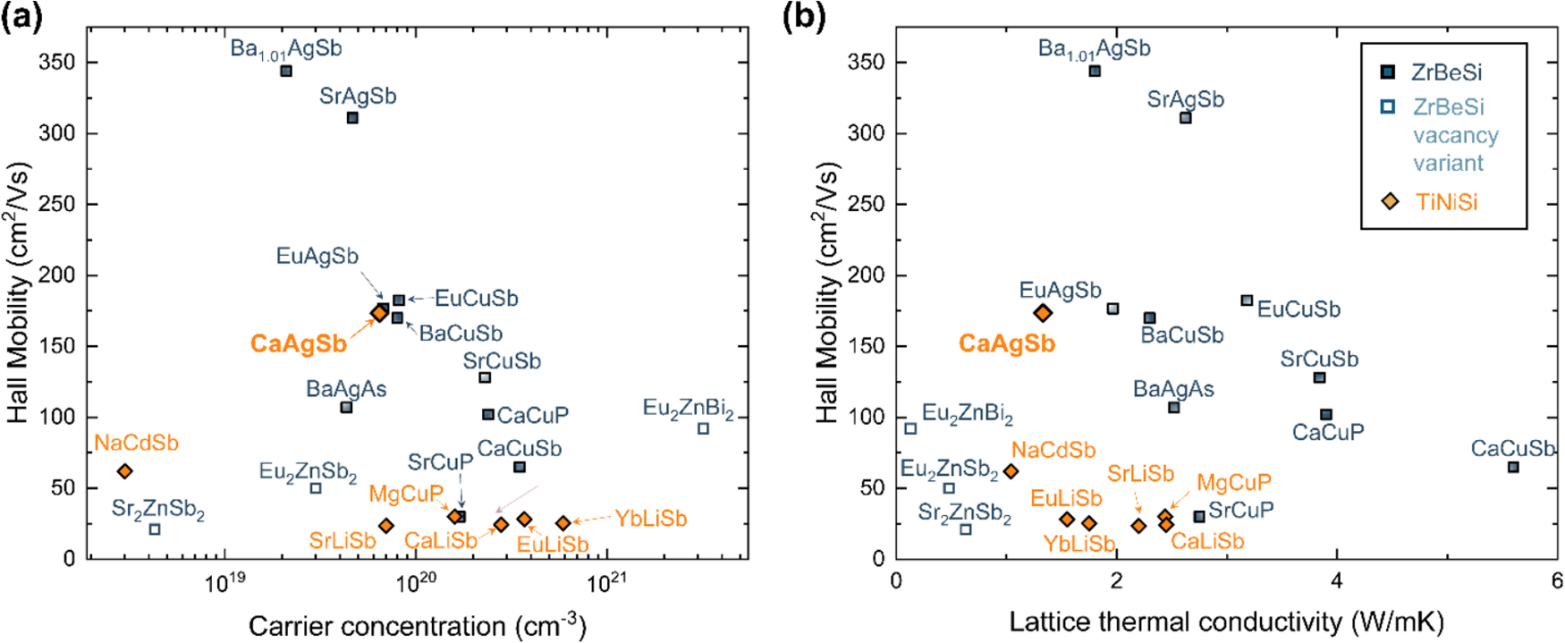
*P*-type carrier mobility in *AMX* Zintl phases as a function of (a) carrier concentration and (b) lattice thermal conductivity. All data is at room temperature. CaAgSb stands out among compounds in the TiNiSi structure as having higher mobility and lower lattice thermal conductivity. However, reduced carrier concentration is still needed to optimize the *zT*.

Zintl compounds are particularly interesting for TE applications due to their intrinsically low lattice thermal conductivities.^4−9^ In particular, the *AMX* (*A* = alkali/alkaline earth/rare-earth metal, *M* = transition metal, *X* = post-transition metalloid) Zintl family has attracted much attention in the past few years.^10−13^*AMX* compounds crystallize in a number of closely related crystal structures, all with promising TE properties.^14^Figure 1 shows a compilation of the room temperature Hall mobility, carrier concentration, and lattice thermal conductivity of *AMX* compounds.^12,13,15−24^ The most prominent of the *AMX* structures is the hexagonal ZrBeSi-structure, in which the *M* and *X* atoms form planar “honeycomb” layers, separated by *A-*cations along the *c*-axis.^25^ Compounds in this crystal structure have been reported to have promising *p*-type TE properties, which is attributed primarily to their high carrier mobility and relatively low lattice thermal conductivity, which can be seen in Figure 1b. There are fewer *AMX* compounds that crystallize in the orthorhombic TiNiSi-structure (Space group *Pnma*), but these have recently garnered attention as well, with at least two compounds reaching *zT* > 1 above 800 K.^20,21^ In general, the orthorhombic modification leads to both lower electronic mobility and lower lattice thermal conductivity. CaAgSb, the subject of the current study, stands out among the orthorhombic phases with hole mobility comparable to some compounds in the ZrBeSi structure type, but still maintaining lower lattice thermal conductivity.^23^

As shown in Figure 1a, *AMX* Zintl compounds tend to be strongly *p*-type, with most compounds exhibiting carrier concentrations of 10^19^–10^21^ holes/cm^3^. In most cases, the “natural” carrier concentration is higher than the optimum for thermoelectric applications, leading many authors to either counter-dope or increase the ratio of cations to anions in an attempt to reduce the carrier concentrations.^26,27^ Unfortunately, reducing the *p*-type carrier concentration in *AMX* Zintl compounds is hampered by their unique tendency to form vacancies on the *M* site.^7^ Indeed, compounds in the ZrBeSi structure have been shown to support up to 50% vacancies on the *M* site.^28^ For example, in EuCuSb, doping with Zn^2+^ at the Cu^1+^ site brings an additional electron into the system. However, to maintain charge balance, a Cu-vacancy is also created. This substitution can be continued until half of the Cu is replaced by Zn while the other half remains vacant,^16^ leading to an overall composition of EuZn_0.5_Sb.^11,29^

In the case of orthorhombic CaAgSb, both experimental efforts to optimize *zT*,^30^ as well as modeling using a single parabolic band (SPB) model, show that the reduction of carrier concentration can lead to improved TE properties.^31^ However, all previous attempts to counter-dope CaAgSb (e.g., with La^3+^ or Zn^2+^) leads to two concurrent effects: a crystallographic transition to the corrugated hexagonal LiGaGe-structure and the formation of compensating Ag-vacancies.^26,30^ It is unclear exactly when the structural transition occurs or what role (if any) Ag-vacancies play in facilitating the phase transition.^30^ This counteracting effect limits the reduction of carrier concentration. Once in the hexagonal structure, further *n*-type doping is always accompanied by a proportional number of vacancies at the Ag-site, which suppresses mobility with little impact on the carrier concentration.^31^ On the other hand, carrier concentration can be *increased* within the orthorhombic structure through alloying.^23^

In this work, we attempted to reduce the carrier concentration in CaAgSb—*without* inducing a transformation to the hexagonal structure type—by using a phase-boundary mapping approach. Our experimental efforts are coupled with defect calculations to understand which defects dominate when CaAgSb is in equilibrium with various competing phases. This approach has been used successfully in several other Zintl compounds to suppress *p*-type defects and optimize carrier concentration, including Mg_3_Sb_2_,^32^ Ca_9_Zn_4.5_Sb_9_,^9^ etc.^19,33^ Ultimately, this work demonstrates the persistence of Ag vacancies and other *p*-type defects in the orthorhombic CaAgSb structure, *regardless* of the local phase equilibrium, and suggests that alternative strategies are still needed to control carrier concentration.

## II Materials and Methods

### II.I Computation

#### (i) Phase Stability

First-principles simulations were performed using density functional theory (DFT)^34,35^ with the projector augmented wave (PAW)^36,37^ method, as implemented in the Vienna Ab Initio Simulation Package (VASP).^38,39^ The exchange–correlation functional was represented by the Perdew–Burke–Ernzerhof (PBE)^40^ formulation within the generalized gradient approximation (GGA),^41^ augmented with a Hubbard *U* correction^42^ (*U* = 5 eV) applied to the 4d states of Ag. Subsequently, the Heyd–Scuseria–Ernzerhof (HSE-06) screened hybrid functional was applied to refine the accuracy of the formation enthalpies, setting the Hartree–Fock exchange mixing parameter to 20%.^43^ The valence electron configurations for Ca, Ag, and Sb were 8, 11, and 5 electrons, respectively. A plane-wave basis set with a cutoff energy of 520 eV was employed.

The thermodynamic stability of each ternary CaAgSb compound was assessed against all known competing phases, including elemental (Ca, Ag, and Sb) and binary phases. The total energies, derived from geometry optimization via first-principles simulations described above, were used to establish the range of chemical potentials (Δμ), ensuring stability of the compound against decomposition into any competing phases. For improved accuracy in the calculation of formation energies of each phase, the Fitted Elemental-phase Reference Energies (FERE) correction formalism was employed.^44^

#### (ii) Defect Calculations

We employed the standard supercell method^45^ to determine the formation energies of native point defects to assess dopability. The formation energy Δ*H*_*D,q*_ of a point defect *D* in charge state *q* is expressed as
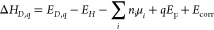
where *E*_*D,q*_ and *E*_*H*_ denote the total energies of the defect supercell and the host supercell without defects, respectively; μ_*i*_ represents the chemical potential of element *i* added (*n*_*i*_ > 0) or removed (*n*_*i*_ < 0) from the host to form the defect; *E*_F_ is the Fermi energy, which varies from the valence band maximum (VBM) to the conduction band minimum (CBM); and *E*_corr_ accounts for the finite size corrections within the supercell approach. The corrections used in evaluating Δ*H*_*D,q*_ included (i) potential alignment corrections and (ii) image charge corrections for charged defects, as detailed by Lany *et al*.^46^ To determine the image charge corrections, we calculated the electronic and ionic dielectric constants using density functional perturbation theory (DFPT) as implemented in VASP.^38^

For defect calculations in CaAgSb, we constructed supercells containing 144 atoms and relaxed the structures using HSE06 to determine the total energies of the supercells. The supercells were relaxed using Brillouin zone sampling with a Γ-centered 2 × 2 × 2 *k*-point grid.

Defect concentration, carrier concentration, and equilibrium Fermi energy were determined by assuming equilibrium defect concentrations and maintaining charge neutrality. The charge neutrality condition is given by
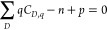
where *q* is the charge state of the defects, *n* and *p* are the free electron and hole concentrations, respectively, and *C*_*D,q*_ is the defect concentration. The concentration *C*_*D,q*_ is given by
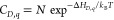
where *N* is the concentration of lattice sites available for defect formation, *k*_B_ is the Boltzmann constant, and Δ*H*_*D,q*_ is the defect formation energy. Carrier concentrations *n* and *p* can be obtained by
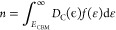

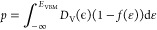
where *D*_C_ (ϵ), *D*_V_ (ϵ), and *f*(ε) are the conduction band density of states, valence band density of states, and the Fermi–Dirac distribution function, respectively.

#### (iii) Crystal Orbital Hamilton Population (COHP)

To explore bonding characteristics within electronic band structures, we employed the crystal orbital Hamilton population (COHP) method,^47,48^ utilizing the Local-Orbital Basis Suite Toward Electronic-Structure Reconstruction (LOBSTER) software package.^49,50^ This method refines the conventional density of states (DOS) by differentiating energies related to bonding and antibonding interactions.

### II.II Synthesis

Eight samples with slight compositional variations (Table S2) from CaAgSb were synthesized using the same process described in ref (23). Pure Sb shots (5N plus, 99.999% purity) were weighed out first based on the stoichiometry Ca_1+*x*_Ag_1+*y*_Sb_1+*z*_ in an argon-filled glovebox. Sb was ball-milled in stainless steel SPEX vials with three 7/16″—stainless steel ball bearings for 10–15 min to create a coating on the vial walls. Then, dendritic Ca (Sigma-Aldrich, 99.9% purity) and Ag shots (Sigma-Aldrich, >99.99% purity) were polished on the surface, cut into small pieces, weighed, and added to the Sb-coated SPEX vial. Each batch contained ∼2 g of starting materials. The mixture was ball-milled for 1 h using a SPEX SamplePrep 8000D mill. After a brief cooling period, the inner vial walls were scraped in a glovebox and the powders were returned for another hour of ball-milling to ensure thorough mixing.

Next, the ball-milled powders were scraped out of the SPEX vials and poured into 10 mm graphite dies for consolidation. Graphite foils were used on each side along with graphite plungers to ensure smooth contact between powders and plungers. The powders were sintered using a Dr. Sinter Spark Plasma Sintering (SPS) press at 1073 K for 10 min under 50 MPa uniaxial pressure. High density pucks with geometric densities >94% were obtained via SPS. The sintered samples were stable in air at room temperature.

### II.III Phase Characterization

SPS’d polycrystalline pucks were polished and characterized using X-ray diffraction (XRD) with Rigaku SMARTLAB diffractometer equipped with a Cu–K_α_ radiation source. XRD patterns are shown using a square root intensity scale to amplify the contributions of the secondary phases. Lattice parameters were determined via Rietveld refinement using the Rigaku PDXL-2 software. Scanning electron microscopy (SEM) and energy dispersive spectroscopy (EDS) were conducted on sample surfaces perpendicular to SPS direction using an AURIGA-Crossbeam Workstation Dual Column SEM. Due to significant overlaps in EDS elemental spectra of Ca, Ag, and Sb, the backscattering electrode (BSE) detector was used to help identify and image secondary phases and compositional variations.

### II.IV Elastic and Transport Properties

Resonant ultrasound spectroscopy (RUS), a nondestructive spectroscopic technique, was used to measure resonant frequencies of dense SPS’d pellets.^51^ The instrumental setup is described by Balakirev *et al*.^52^ The open-source RUSpy software was used to collect the spectra and RUSCal software was used to extract the elastic constants by inverse numerical analysis.^53^ The isotropic model was used to calculate two independent elastic constants, *c11* and *c44*, and the longitudinal and shear sound velocities.^53^

Thermal diffusivity was measured using a NETZSCH Light Flash Apparatus (LFA) 467 HyperFlash. The equation *κ = ϱ × D × C*_*v*_ was used to calculate the total thermal conductivity, *κ*, where *ϱ* is the geometric density, *D* is thermal diffusivity and *C*_*v*_ is the heat capacity calculated using the Dulong–Petit approximation.^54^ Electrical resistivity and Hall effect measurements were conducted under dynamic vacuum on a custom-built apparatus with van der Pauw geometry.^55^ The Seebeck coefficients were measured on a custom apparatus under 300 Torr N_2_ atmosphere.^56^ Each measurement is assumed to have an uncertainty of approximately 5%, resulting in an estimated uncertainty of ∼20% in reported *zT* values.^57^

## III Results

### (a) Theoretical Phase Stability and Defects

**Figure 2:**
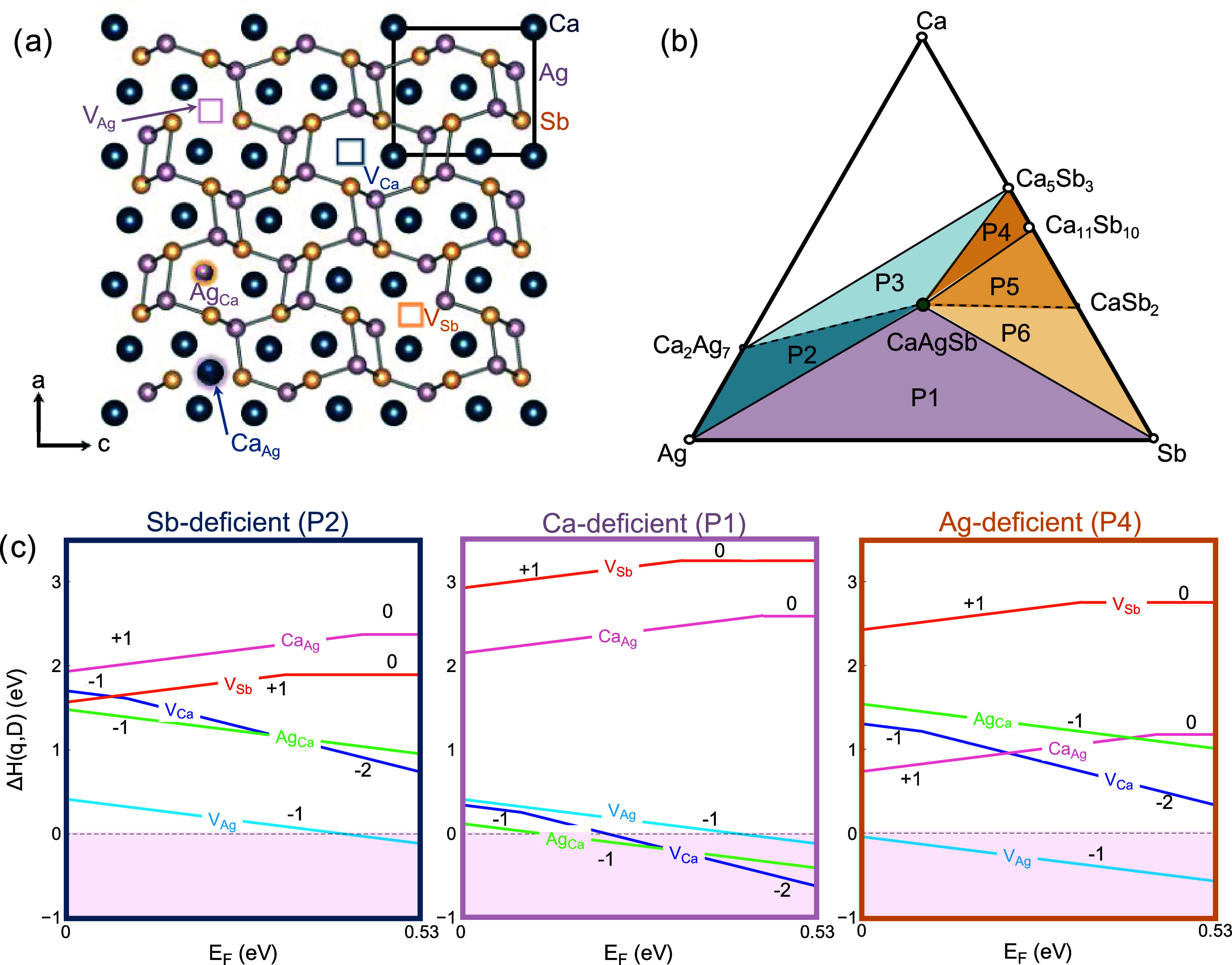
(a) CaAgSb crystallizes in the orthorhombic TiNiSi-type structure (Space group—*Pnma*). The formation energies of various point-defects (as shown) were investigated in this study to determine what controls *p*-type conduction in CaAgSb. (b) The Ca–Ag–Sb phase space contains six three-phase regions around CaAgSb. (c) Defect formation energy diagrams reveal that acceptor type defects are dominant under all equilibrium conditions.

First, the stability of different competing phases was checked using convex hull analysis calculated at different levels of density functional theory. Figure S1 shows the chemical potential diagrams calculated using different approximations to the exchange correlation potential. CaAgSb, structure shown in Figure 2a, is a small band gap semiconductor. As such, the gap closes when DFT calculations use the PBE exchange correlation functional.^40^ Incorporating the Hubbard parameter within the PBE+*U* approximation on Ag 4d-orbitals yielded a band gap of 0.09 eV.^42^ The hybrid exchange–correlation functional HSE-06 predicted the band gap to be 0.53 eV.^43^ The chemical potential diagrams and electronic density of states calculated through all three levels of theory are shown in Figures S1 and S2, respectively. The predicted stability of the Ca–Ag and Ca–Sb binaries varies, depending on the level of theory.

Calculations using PBE+*U* came closest to the reported experimental phase diagram, in which binary phases Ca_2_Ag_7_ and CaSb_2_ are reported to form. However, the *U* values employed were fixed for all compounds in the Ca–Ag–Sb phase space, which is not necessarily realistic. Based on the band gap and lattice parameters of the relaxed crystal structures (Table S1), we concluded that hybrid functionals most accurately capture the experimentally observed properties of CaAgSb. Therefore, the results from HSE-06^58^ were used to calculate defect formation energies, as outlined in the Methods section. The native defects include vacancies *V*_Ca_, *V*_Ag_, *V*_Sb_, and antisite defects Ca_Ag_ and Ag_Ca_, all of which were considered in varying charge states. No reasonable interstitial sites were identified. By convention, we only show the minimum energy charge state of each defect in Figures 2c and S3. The relevant defects are visualized in Figure 2a. Due to symmetry-constraints within the TiNiSi-structure, each atom has only one unique coordination environment.

Six different regions of three-phase equilibria (or ternary invariant points) were predicted around the line compound CaAgSb using hybrid functionals, as shown in Figure 2b. Defect formation energies calculated for all of the three-phase regions are shown in Figure S3. Our discussion will focus primarily on the three regions shown in Figure 2c, which highlight Sb-deficient (P2), Ca-deficient (P1), and Ag-deficient (P4) growth conditions. In almost all growth conditions, *V*_Ag_ is the dominant defect, exhibiting the lowest formation energies for all Fermi energies from the VBM to the CBM, ensuring strong *p*-type character. This is surprising for two reasons; first, in many Zintl phases, cation-rich conditions can reduce the energy of *n*-type defects.^7,32,59^ Here, however, *n*-type defects like *V*_Sb_ and Ca_Ag_ exhibit very high formation energies and are unlikely to form irrespective of growth conditions. Therefore, *p*-type behavior can be expected in CaAgSb across the full stability region. Second, in most Zintl phases, vacancies on the *A* site have significantly lower energy than vacancies on the transition metal or main group metal *M* site.^9,10,32,60^ In CaAgSb, however, Ca vacancies (*V*_Ca_), along with antisite defects (Ag_Ca_) are observed to become competitive with *V*_Ag_*only* in Ca-deficient conditions, as can be seen in region P1 in Figure 2c.

**Figure 3:**
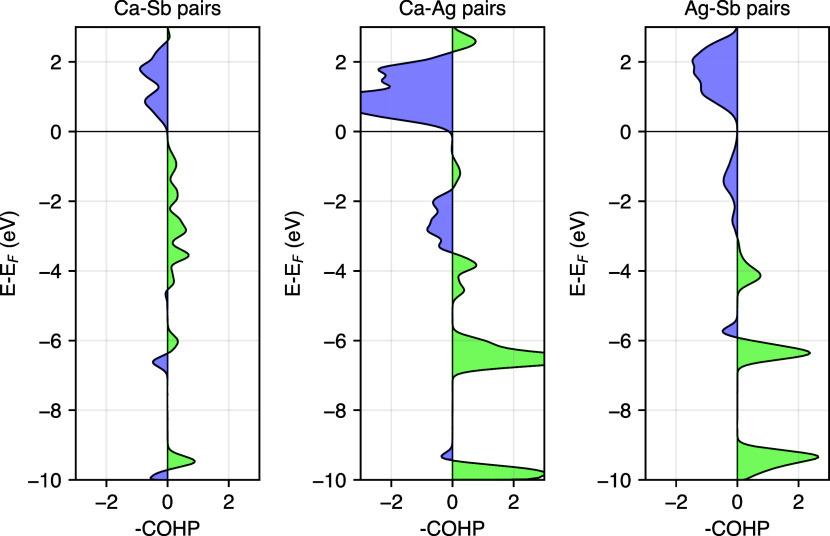
Crystal Orbital Hamilton Population (COHP) analysis for Ca–Sb, Ca–Ag, and Ag–Sb bonds in CaAgSb, showing bonding orbital-pair interactions to the right and antibonding interactions to the left.

To gain insights into the low formation energy of Ag-vacancies, the crystal orbital Hamilton population (COHP) was calculated for Ca–Sb, Ca–Ag, and Ag–Sb pairs (See Figure 3). COHP elucidates the bonding and antibonding character of electronic states in a crystal, which can provide insight into bond strength. In CaAgSb, the Sb atoms form distorted tetrahedral coordination around the Ag atoms, and each tetrahedron has one edge-sharing and one corner-sharing neighbor, respectively. Our COHP calculations show that the antibonding Ag–Sb orbitals from the tetrahedra make up the uppermost valence bands. Similar observations were made in CuInTe_2_ and related diamond-like chalcopyrite systems. Xi et al. reported that the *E*_F_ lies above the antibonding Cu–Te orbitals,^61^ while Adamczyk et al. noted that *V*_Cu_ remains a low energy defect in CuInTe_2_ under all growth conditions.^62^ Such filled antibonding states might be correlated to high energy, unstable bonds that are easy to break, and may be the reason for the low formation energy of Ag vacancies in CaAgSb. Furthermore, the dominance of Ag-vacancies across all chemical potentials suggests that the thermodynamically stable stoichiometry could be *AM*_*1–*δ_*X*, where *δ* represents the number of Ag-vacancies.

To predict the carrier concentration resulting from the native defects in CaAgSb at each ternary invariant point, the associated equilibrium Fermi energy level was calculated using the charge neutrality condition as described in the Methods section. The predicted equilibrium *E*_F_ lies below the VBM across all temperatures for all regions, except in the Ag-rich P2 and P3 (Figure S4), where *E*_F_ is close to the VBM edge. In the regions labeled P1, P4, P5, and P6, the predicted carrier concentration is on the order of ∼10^19^ to 10^20^ cm^–3^ at room temperature and reaches around ∼10^20^ to 10^21^ cm^–3^ at higher temperatures, showing degenerate behavior. In the Ag-rich P2 and P3 regions, *E*_F_ is expected to lie approximately 0.05–0.08 eV above VBM at room temperature, corresponding to lower predicted carrier concentration of ∼10^17^ cm^–3^. Even in P2 and P3, however, the equilibrium *E*_F_ is predicted to cross into the valence band with increasing temperature, leading to carrier concentrations above 10^19^ cm^–3^, as shown in Figure S4b. A single parabolic band model predicts a *zT* of 1 at carrier concentrations of ∼10^19^ cm^–3^.^23^ Therefore, regions P2 and P3 appear to be the most promising to optimize the electronic properties of CaAgSb by reducing the carrier concentration.

### (b) Experimental Phase Stability

**Figure 4:**
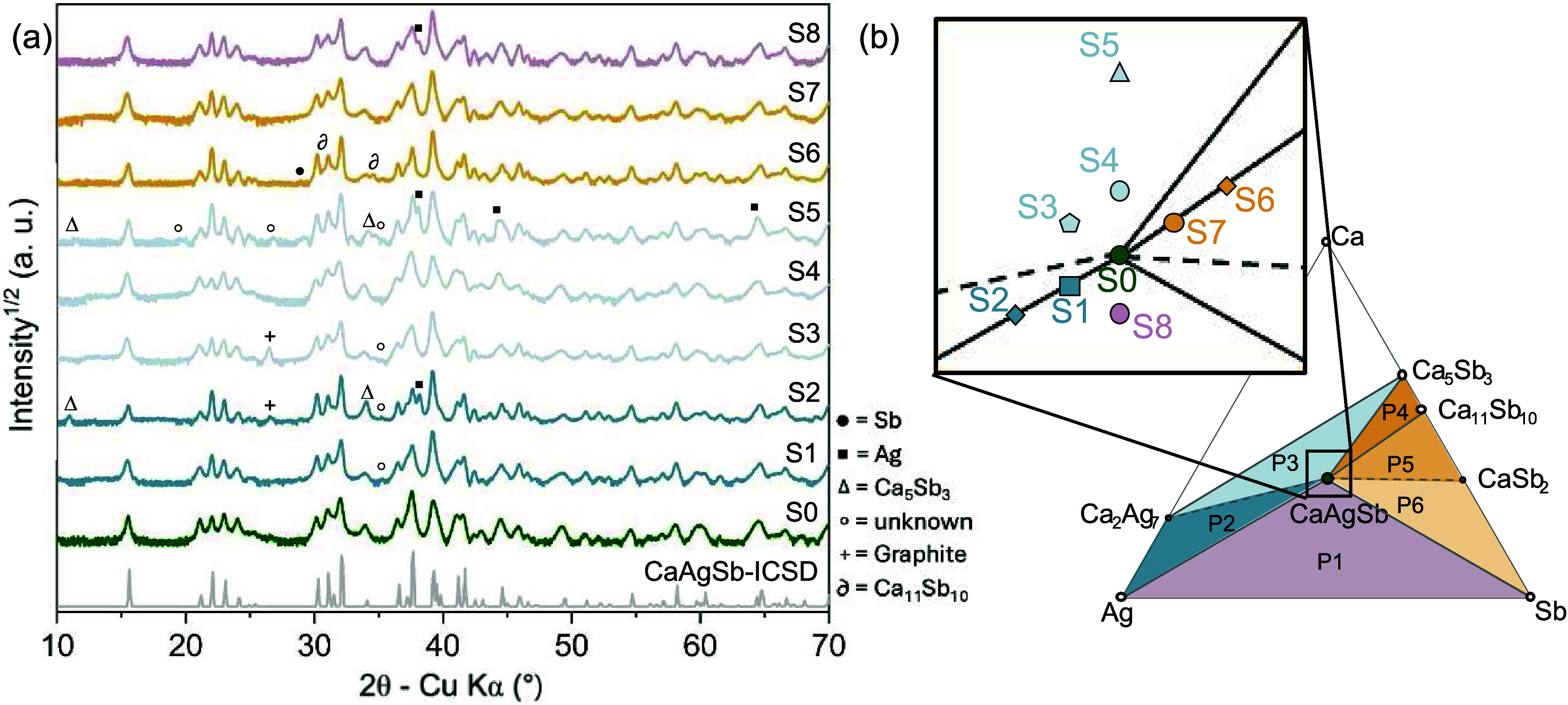
(a) Powder XRD patterns on SPS’d pucks reveal secondary phases that are used to confirm the positions of samples in ternary phase space. Note that the graphite peaks come from remnants of the graphite-foil used during SPS. (b) The nominal positions of samples S1–S8 in the Ca–Ag–Sb ternary phase space. S0 represents the reference CaAgSb sample from our previous work.

Eight off-stoichiometry CaAgSb samples, labeled S1–S8 were synthesized for this study. The nominal compositions are listed in Table S2 and their positions on the ternary phase diagram are shown in Figure 4b. The powder X-ray diffraction patterns are shown in Figure 4a, where square-root of relative intensity is shown to amplify the peaks of the secondary phases. The primary phase in all eight samples is orthorhombic CaAgSb, and Rietveld refinements show no significant or systematic changes in lattice parameters. The samples within 5 at. % deviation from the 1:1:1 stoichiometry (S1, S3, S4, S7, and S8) were nearly phase-pure, based on the XRD patterns. The main exception is sample S8 (CaAg_1.05_Sb_1.05_), which exhibits a clear elemental Ag peak. However, BSE-SEM revealed impurities that could not easily be detected via XRD. For both S1 (CaAg_1.05_Sb) and S4 (Ca_1.05_AgSb), the phase contrast in BES-SEM images suggests the presence of Ca-deficient and Ca-rich phases, respectively (see Figure S5). For samples with greater than 10 at. % deviation in composition from CaAgSb, significant peaks arising from secondary phases could be observed in the XRD patterns. Elemental Ag peaks were observed in samples S2 (CaAg_1.10_Sb), S5 (Ca_1.15_AgSb), and S8 (CaAg_1.05_Sb_1.05_), and Ca_5_Sb_3_ peaks were observed in S2 and S5. The phase contrast in BES-SEM imaging gave further evidence of the presence of these secondary phases.

Overall, reasonable agreement was found between the observed secondary phases and the predicted phase diagram, except in one regard. We did not observe the binary phases, Ca_2_Ag_7_ (expected in samples S1–S5) or CaSb_2_ (expected in S6 and S7) in any of our samples. In sample S5, for example, both Ag and Ca_5_Sb_3_ were observed in the XRD pattern, whereas Ca_2_Ag_7_ and Ca were predicted. This suggests that under our synthesis conditions, Ca_2_Ag_7_ may have failed to form due to kinetic limitations. It is also possible that elemental Ca and Ca_2_Ag_7_ were present in the sample after SPS but decomposed in air during handling prior to XRD measurements. Finally, we note that an unidentified peak was observed at ∼35.2° in samples S1, S2, S3, and S5, which does not belong to any of the predicted compounds or respective oxides. Several unidentified peaks were seen in the S5 sample (19.6, 26.85, 29.2, 32.9°), which suggests that more undiscovered phases may still exist in this ternary space. However, despite some uncertainty in the exact phase makeup of the samples, the presence of Ag, Ca_5_Sb_3_, and/or Ca_11_Sb_10_ impurities confirms that the samples exist in Ag-deficient, Ca- deficient, and Sb- deficient regions of the phase diagram, as intended.

### (c) Electronic and Thermal Transport Properties

**Figure 5:**
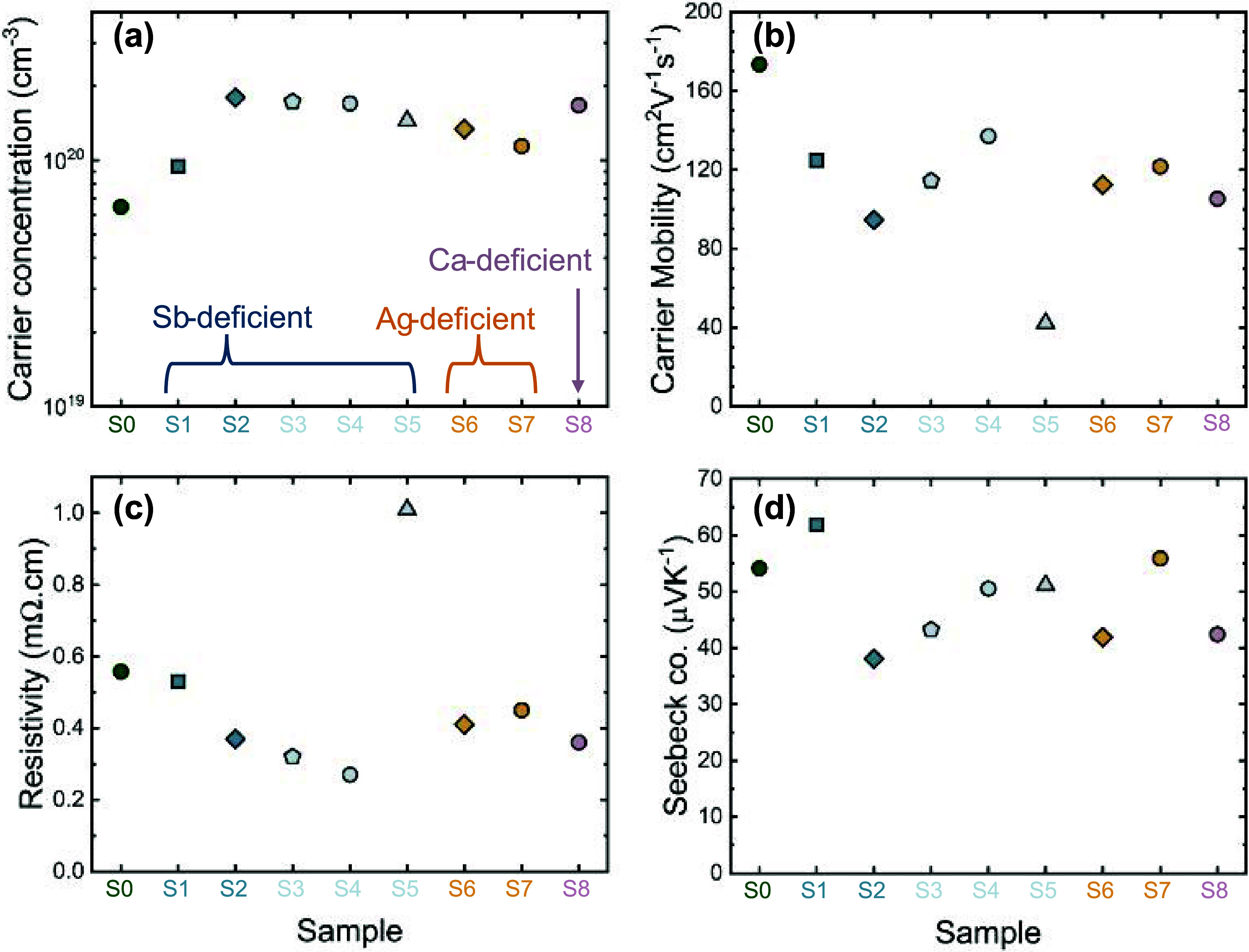
At 323 K, (a) carrier concentration is found to be within a narrow range of between 0.6–1.8 × 10^20^ cm^–3^, while the (b) carrier mobility decreases with increasing secondary phases. These electronic transport properties result in small changes in both (c) resistivity and (d) Seebeck coefficient as *E*_F_ remains largely pinned.

**Figure 6:**
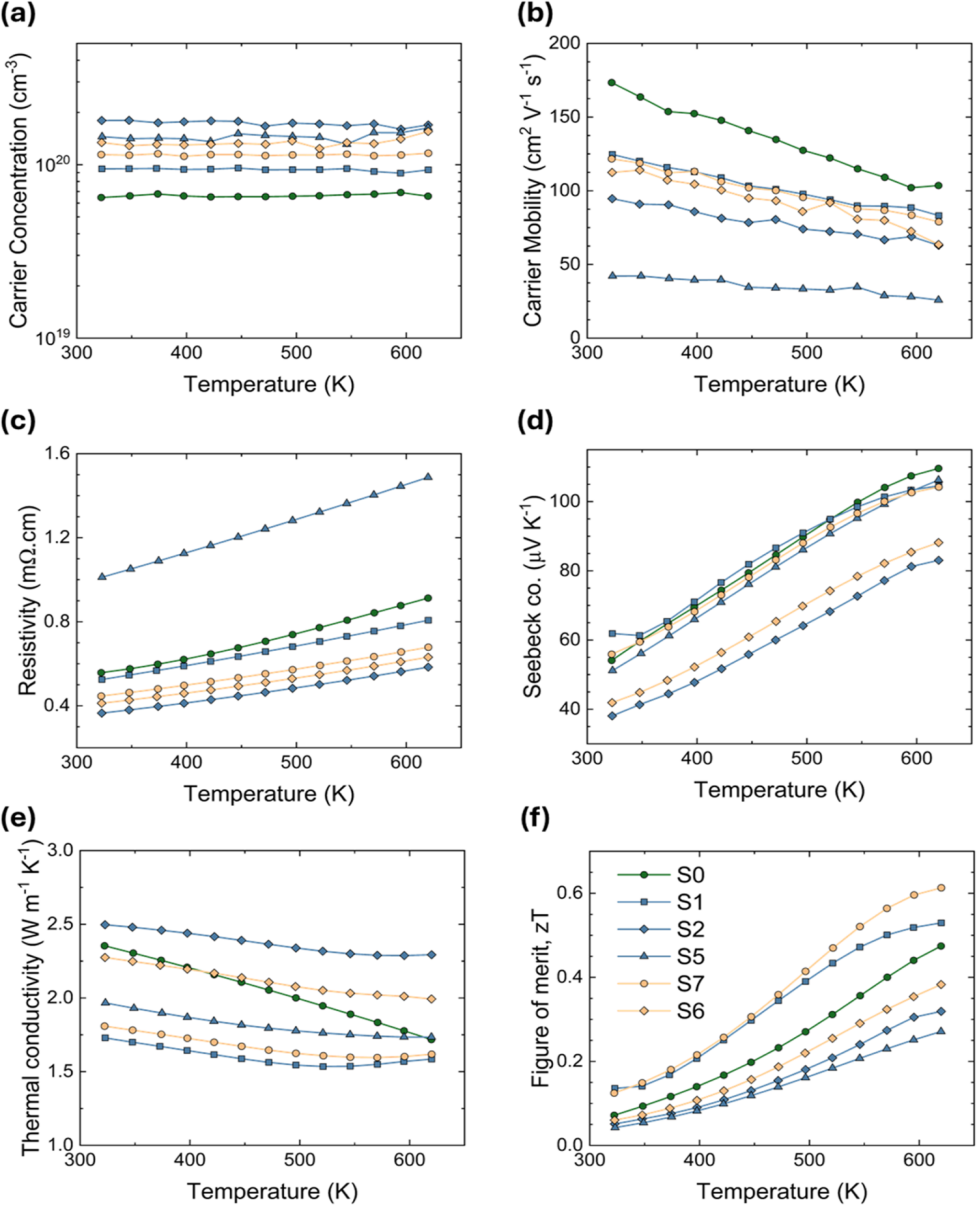
With increasing temperature, (a) carrier concentration remains constant while (b) carrier mobility decreases. All samples exhibit degenerate-like behavior as apparent from increasing (c) resistivity with temperature. (d) Seebeck coefficient increases with increasing temperature and begins to flatten out near 600 K, where bipolar conduction may begin. (e) All samples exhibit similar total thermal conductivity, culminating in little improvement in (f) *zT*. The data reported here are from the heating cycles for each measurement, since no apparent hysteresis was observed between heating and cooling.

Transport properties at 323 K for all samples (S1–S8) encompassing different phase spaces are shown in Figure 5. All of the samples were found to exhibit *p*-type behavior, and the compositional variation was found to have very little impact on the magnitude of the carrier concentration. The carrier concentration remains within a narrow window of 6.0 × 10^19^ and 1.8 × 10^20^ cm^–3^, suggesting that the Fermi energy is pinned slightly below the VBM. All of the samples were found to have carrier concentration slightly higher than the nominally stoichiometric sample, S0, reported in our prior work.^23^ As shown in Figure 6a, in all samples, the *p*-type carrier concentration is constant as a function of temperature, consistent with the behavior of an extrinsic semiconductor. Little variation was found in the magnitude or temperature dependence of the Seebeck coefficients across the series of samples, which is also consistent with the narrow range of carrier concentrations. Overall, these results are consistent with our theoretical predictions that Ag vacancies are the dominant defect, pinning the Fermi level in, or near, the VBM, regardless of whether CaAgSb is synthesized in Ag-rich, Sb-rich, or Ca-rich conditions.

The carrier mobility of the samples (Figure 5b) can be correlated with the impurity concentrations, with the lowest mobility found in samples with the greatest deviation from the 1:1:1 stoichiometry. All of the samples have lower mobility than that in the nominally stoichiometric reference sample, S0. For most samples, the resistivity is slightly lower than that of the nominally stoichiometric CaAgSb sample, due to their higher carrier concentrations. Sample S5 (Ca_1.15_AgSb) is an exception, which has much higher resistivity stemming from its low mobility. As shown in Figure 6b, the carrier mobility of all samples decreases with increasing temperature. However, temperature-dependence of the mobility is significantly flattened for the samples with the highest impurity concentrations, for example S5 and S2, likely due to increased point defect scattering and grain boundary resistance.

The variation in total thermal conductivity of the CaAgSb samples, shown in Figure 6e, can be attributed to variation in both the electronic (*κ*_E_) and lattice (*κ*_L_) terms. These contributions are plotted separately in Figure S7. Several features of the lattice thermal conductivity trends are surprising. First, the *κ*_L_ values show a large spread, ranging from 0.34 to 1.32 W/mK at 323 K. The sample with the highest fraction of impurity phases, S5, appears to exhibit higher lattice thermal conductivity than any other sample across the entire temperature range. The sound velocity of this sample is comparable to the other samples (see SI Table 2). Therefore, given the similar speed of sound and presumably increased concentration of scattering centers, it is strange that sample S5 exhibits much higher lattice thermal conductivity than the pristine CaAgSb. The possibility of secondary phases having higher thermal conductivity is a possible suspect. On the other end of the *κ*_L_ spectrum, samples S1 and S7 both exhibit extremely low lattice thermal conductivity across the entire measured temperature range. Overall, we find that differences in impurity phase concentration cannot explain the differences in *κ*_L_. Further, no obvious correlation between electronic mobility and lattice thermal conductivity was observed, indicating that the electronic carrier scattering and phonon scattering are not controlled by the same microstructural features or point defects.

As we have described previously, the combination of high electronic conductivity and low lattice thermal conductivity in CaAgSb makes calculating *κ*_L_ fraught. Because *κ*_E_ is large, and *κ*_L_ is small, even a small overestimation of *κ*_E_ can yield physically impossible negative *κ*_L_. Indeed, in the present study, if we use temperature-dependent Lorenz numbers obtained from a standard SPB model,^63^ we obtain negative *κ*_L_ for the most electrically conducting samples. This is, of course, impossible. From our prior work on the CaAgSb_1–*x*_Bi_*x*_ system, it is clear that in samples with complex electronic band structure and low lattice thermal conductivity, the Weidemann-Franz law and simple Lorenz number models often fail to capture the true physics behind thermal transport properties.^64^ Recent years have seen an increase in the number of “negative” thermal conductivity reports for some promising TE material candidates.^19,65,66^ Electron–phonon interactions and subsequent coupling may play a significant role in these compounds and no straightforward explanation is available in literature to the best of our knowledge. In our prior work, we tested various models for the Lorenz number in the CaAgSb_1–*x*_Bi_*x*_ system and concluded that Lorenz number may extend below the nondegenerate limit at high temperatures.^23^ Our modified-Landauer approach, where a combination of computations and experiments was employed, was more successful in modeling electronic thermal conductivity in CaAgSb than the SPB model.^23^ For this reason, the previously calculated *L*_Landauer_ was used in the current study. Regardless, the unusual variation in *κ*_L_ values for the present series of samples suggests that there is more work to be done to accurately separate the contributions of *κ*_E_ and *κ*_L_ in systems with high electrical conductivity and low lattice thermal conductivity. It is also worth noting that we observed up to 20% anisotropy in tensorial, direction-dependent, transport properties in our previous work.^23^ This anisotropy was a consequence of SPS pressing direction, and readers are advised to take it under advisement when interpreting these results.^67^

Finally, with respect to the figure of merit, *zT*, we find a relatively large range of values in this series of samples. The spread in *zT* is not caused by variations in carrier concentrations or Seebeck coefficient, both of which are largely unaffected. Ultimately, a trade-off between reduced mobility and decreased lattice thermal conductivity dictates the final *zT* values. Compared with our nominally stoichiometric sample, S0, the samples in the current study with the lowest κ_L_, S1 (CaAg_1.05_Sb) and S7 (Ca_1.05_AgSb_1.05_), show the largest improvement in *zT*. The Ag-deficient sample S7 (Ca_1.05_AgSb_1.05_) exhibits a maximum *zT* of 0.61 at 623 K. 10 atom % or higher deviation from the stoichiometric composition CaAgSb leads to lower mobility and therefore poorer *zT*. Although the defect chemistry limits carrier tunability in the otherwise promising thermoelectric material CaAgSb, additional routes could be explored to improve the TE properties. Isovalent alloying with As at the Sb site might increase in the band gap, which could in turn lead to reduced defect energies and lower carrier concentrations. According to the SPB model predictions, this could lead to improved *zT*.

## IV Conclusions

This work elucidates the carrier tunability in the promising thermoelectric material CaAgSb using a phase-boundary mapping approach. Despite minimum deviation in composition, CaAgSb remains predominantly p-type with a narrow window of carrier tunability. Through a combination of computational and experimental results, we show that the carrier concentration, and therefore *E*_F_, remains pinned due to the tendency of CaAgSb compound to form *V*_Ag_ defects. This is in contrast to other Zintl families, where *A*-cation vacancy typically is the “killer defect”. Incorporating excess Ag does not change the electronic picture as the excess Ag simply sediments out as secondary phases. However, minimum mobility loss and an overall low thermal conductivity leads to an improved *zT* of 0.61 at 623 K.

## Supporting Information Available

Chemical potential diagrams and electron density of states calculated using three different exchange correlation functionals, defect energy diagrams, SEM backscattering images, lattice parameters, speed of sound, representative Rietveld refinements, and the lattice and electronic contributions to thermal conductivity (PDF)
