# Supplementary material for: Ag Vacancies as “Killer-Defects” in CaAgSb Thermoelectrics
Source: ACS Appl Energy Mater. 2025 Feb 11;8(4):2318–27. doi: 10.1021/acsaem.4c02907 (PMC11863183; doi:10.1021/acsaem.4c02907)
Supplement: Supplementary file 1 — ae4c02907_si_001.pdf [file ae4c02907_si_001.pdf]

Supporting Information

Ag vacancies as ‘killer-defects’ in CaAgSb thermoelectrics

A. K. M. Ashiquzzaman Shawon[1], Ferdaushi Alam Bipasha[2], Channyung Lee[2], Kamil M. Ciesielski[3], Brian Tijan[1], Eric S. Toberer[3], Elif Ertekin[2], Alexandra Zevalkink[1]*

[1] Department of Chemical Engineering and Material Science, Michigan State University, East Lansing, Michigan 48824, United States

[2] Department of Mechanical Science and Engineering, University of Illinois at Urbana-Champaign, Urbana, Illinois 61801, United States

[3] Department of Physics, Colorado School of Mines, Golden, Colorado 80401, United States

*Corresponding author: alexzev@msu.edu

SI Section 1: Computed chemical potentials and electron density-of-states (DOS)

Phase stability predictions were made using convex hull with three different DFT functionals. Figure S1 shows the chemical potential diagrams calculated using PBE, PBE+U, and HSE-06 functionals. Relaxing the orthorhombic CaAgSb structure reported in ICSD (Coll. Code – 56982) using different DFT functional resulted in varied lattice parameters, as shown in Table S1. The lattice parameters and unit cell volume determined using HSE-06 functionals agree the most with experimentally-obtained values. Electron density of states were calculated following self-consistency calculations and the results are shown in Fig S2.

Table S1: Comparison of computed lattice constants using DFT-PBE, DFT-PBE+U and DFT-HSE-06 with experimental values.

Calculation method	a (Å)	b (Å)	c (Å)	Difference in a (%)	Difference in b (%)	Difference in c (%)	Volume (Å^3)
Experiment	7.75	4.60	8.42	0.0	0.0	0.0	300.0
PBE	7.72	4.62	8.49	-0.3	0.5	0.9	303.0
HSE-06	7.69	4.59	8.50	-0.8	-0.3	0.9	299.6
PBE+U	7.74	4.61	8.55	0.0	0.4	1.5	305.5

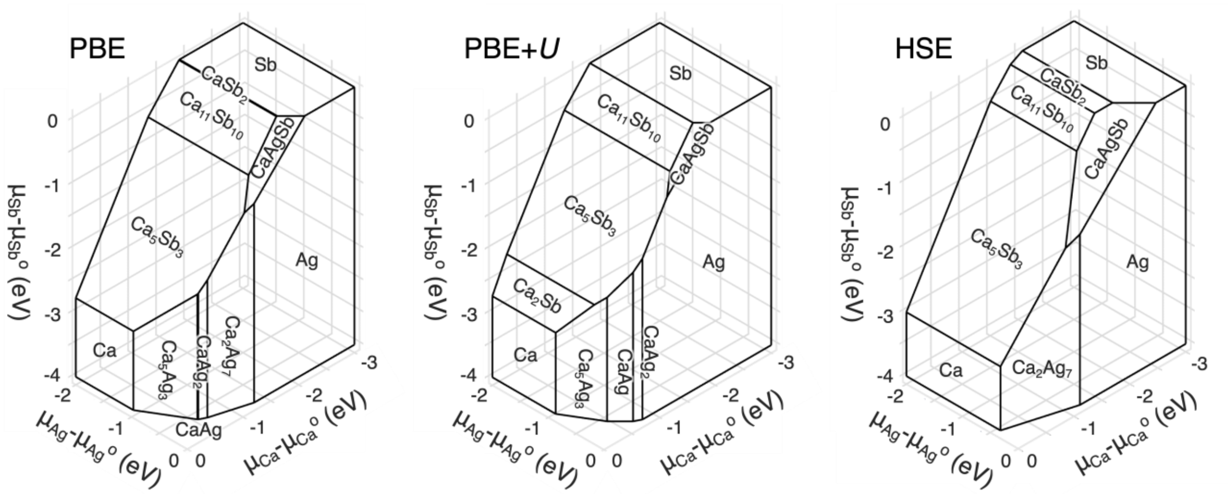

Figure S1: Chemical potential phase diagram, showing the region of stability of CaAgSb with respect to the elemental chemical potentials across three different levels of theories.

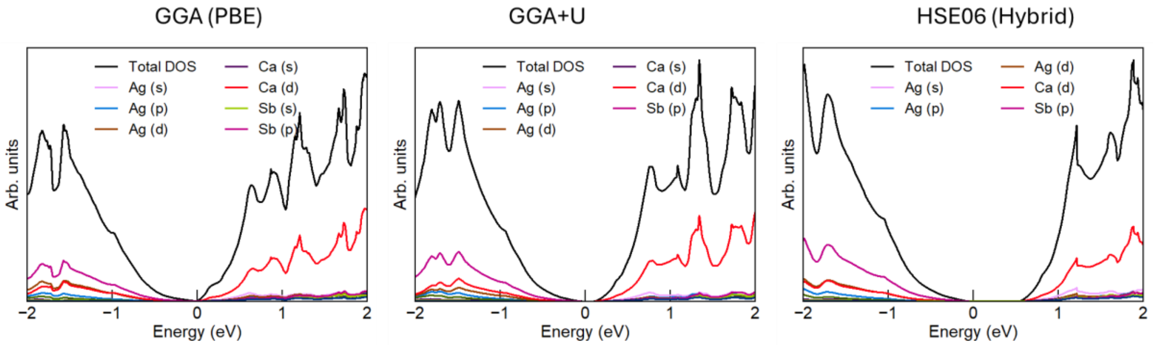

Figure S2 Computed DOS for CaAgSb using DFT-PBE, DFT-PBE+U and DFT-HSE-06 functionals.

SI Section 2: Defect energy diagrams

Defect formation energies for native defects in CaAgSb were calculated using the approach described in Methods. The chemical potential diagrams calculated using HSE-06 functionals were used to calculate defect energy diagrams under different chemical spaces. Figure S3 shows the defect energy diagrams with different competing phases for all growth condition labelled P1 – P6.

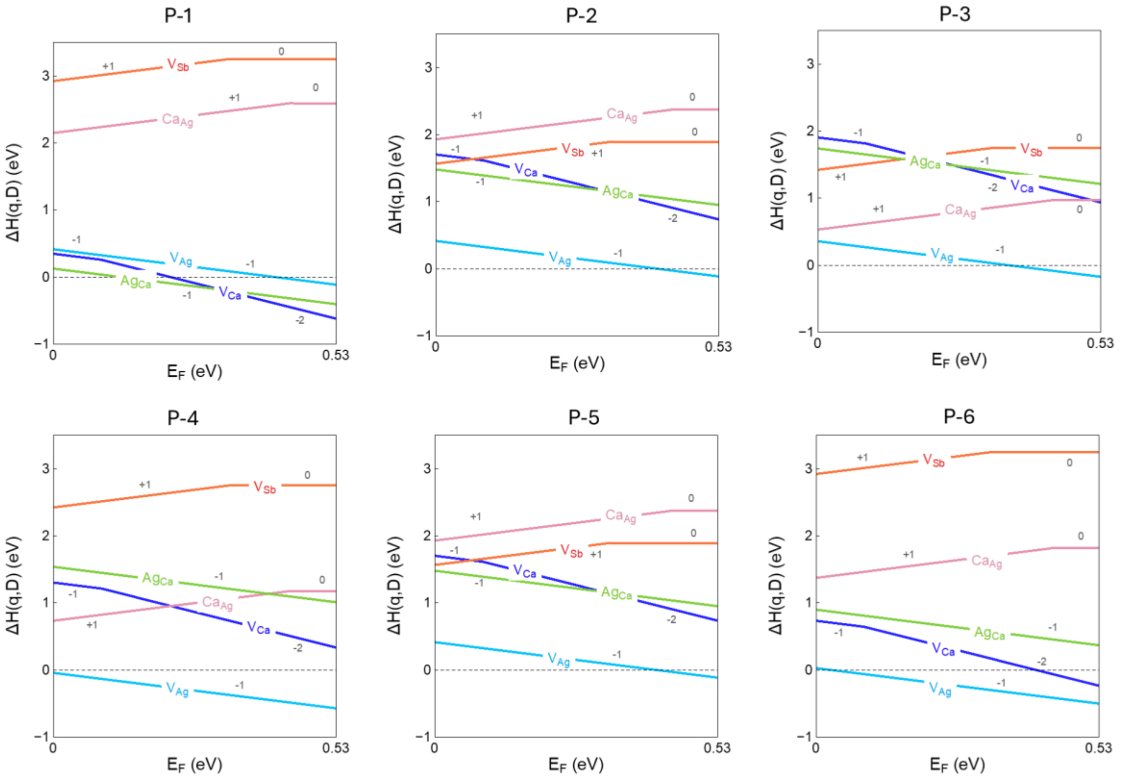

Figure S3: Defect formation energy for native point defects as a function of Fermi level (E_F) in CaAgSb under all growth conditions with different chemical spaces with respect to the elemental chemical potentials computed using DFT-HSE functionals.

SI Section 3: Computed transport properties and bonding behavior

Equilibrium Fermi energy and carrier concentration were obtained from the defect formation energies using the charge neutrality condition described in Methods. Similar to the defect diagrams, computed E_F and carrier concentration are shown for all growth conditions from P1 to P6.

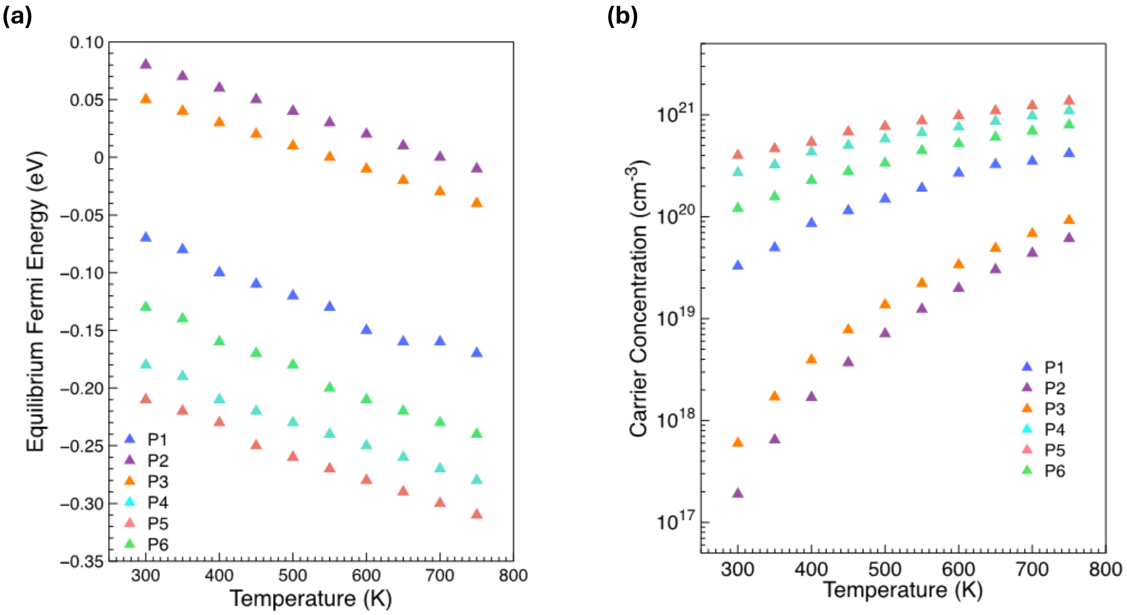

Figure S4: Prediction of (a) equilibrium Fermi energy and (b) net hole carrier concentrations as a function of temperature throughout the stability regimes in CaAgSb showing intrinsic *p*-type to degenerate *p*-type behavior in all growth conditions.

SI Section 4: SEM imaging using backscattering electrons:

Backscattering images are shown for select samples as Fig S5. In the BSE mode, color contrast represents density differences that are caused by phase separation. Darker color represents lower density, while brighter color represents higher density. For comparison, CaAgSb has a density of 6.02 g/cm^3 while Ca_5Sb_3 has a density of 3.79 g/cm^3. Ag has a density of 9.88 g/cm^3 and can be seen as bright spots in BSE images. Although accurate elemental compositions could not be determined due to x-ray peak overlap, dark spots were found to be significantly Ag-poor while the bright regions were predominantly Ag-rich.

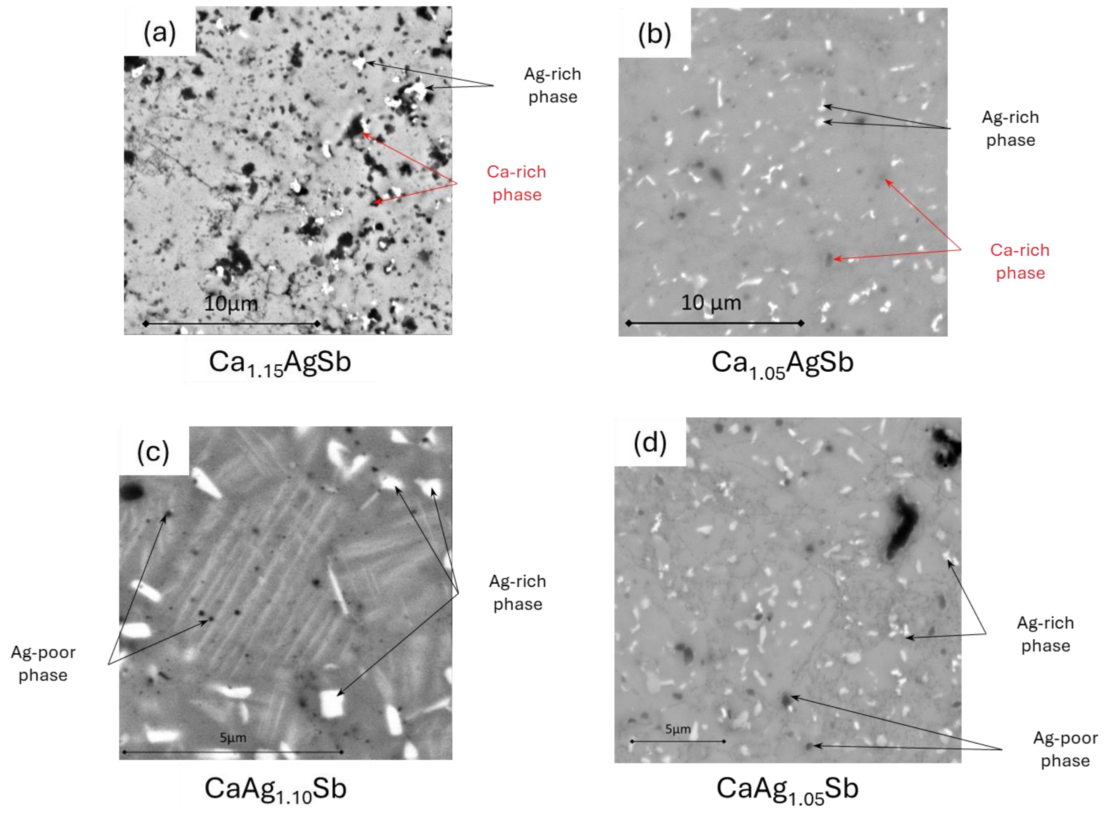

Figure S5: Backscatter electron imaging done on (a) S5 ($Ca_{1.15}AgSb$), (b) S4 ($Ca_{1.05}AgSb$), (c) S2 ($CaAg_{1.10}Sb$) and (d) S1 ($CaAg_{1.05}Sb$). Dark (bright) regions represent secondary phases with lower (higher) density than CaAgSb (6.02 g cm^{-3})

SI Section 5: Lattice parameters, thermal, and elastic properties

Table S2 lists the experimental lattice parameters, densities, and sound velocities for all samples. Lattice parameters were calculated through Rietveld refinement of XRD patterns. Figure S6 shows representative Rietveld refinements for select samples. Relative densities were calculated on the basis of the theoretical density (6.02 g/cm^3) for pristine CaAgSb. Some relative densities are >100% due to the density of secondary phase Ag being much higher than that of CaAgSb. v_L and v_T are the longitudinal and transverse speeds of sound respectively.

Table S2: Nominal compositions of all samples in this study along with select properties at 300 K:

Sample number	Nominal composition	a (Å)	b (Å)	c (Å)	Volume (Å)^3	Geometric density (g/cm^3)	Relative density	v_L (m/s)	v_T (m/s)
S0	CaAgSb	7.7482	4.5977	8.4208	299.98	5.93	98.4	3969	2164
S1	$CaAg_{1.05}Sb$	7.698(12)	4.587(7)	8.39(2)	296.26	5.92	98.2	4017	2192
S2	$CaAg_{1.10}Sb$	7.693(12)	4.585(8)	8.395(19)	296.11	6.10	101.2	3936	2102
S3	$Ca_{1.05}Ag_{1.05}Sb$	7.693(15)	4.587(9)	8.38(2)	295.71	6.04	100.1	4003	2145
S4	$Ca_{1.05}AgSb$	7.696(13)	4.570(11)	8.43(3)	296.49	5.95	98.6	4032	2176
S5	$Ca_{1.15}AgSb$	7.717(18)	4.58(2)	8.37(6)	295.83	5.69	94.4	3978	2192
S6	$Ca_{1.10}AgSb_{1.10}$	7.686(14)	4.585(8)	8.42(4)	296.72	5.95	98.6	4025	2190
S7	$Ca_{1.05}AgSb_{1.05}$	7.69(2)	4.569(14)	8.40(7)	295.14	5.96	98.9	4008	2189
S8	$CaAg_{1.05}Sb_{1.05}$	7.687(13)	4.585(8)	8.43(3)	297.11	6.05	100.3	3999	2136

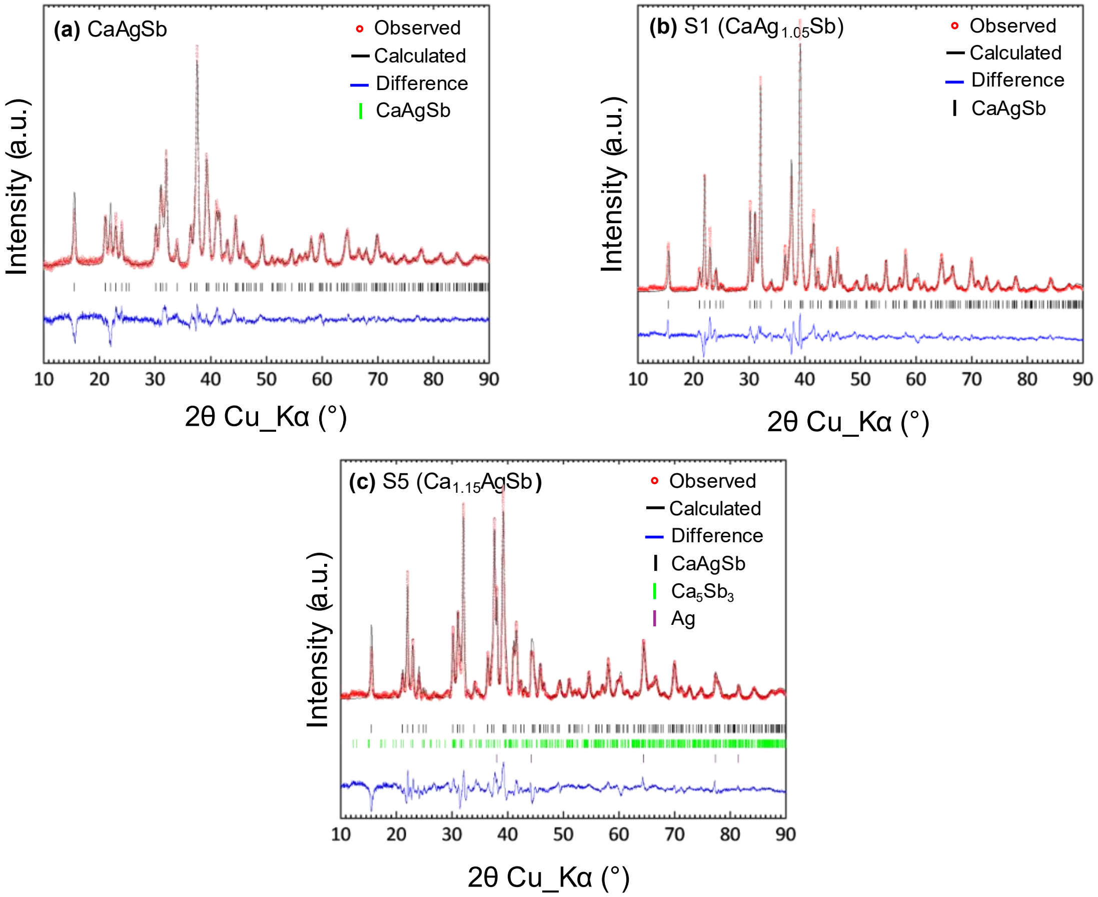

Figure S6: Representative Rietveld refinements are shown for samples (a) stoichiometric CaAgSb sample, (b) sample S1, and (c) sample S5.

SI Section 6: Lattice and electronic contributions to thermal conductivity

Electronic contributions to thermal conductivity, κ_E, were calculated using the Weidemann-Franz law, $\kappa_E = (L*T)/\rho$, where T is absolute temperature, ρ is the resistivity, and L is the Lorenz number. The Lorenz number derived from the single parabolic band model results in negative κ_E in some samples. In our previous work, we detailed limitations of the Lorenz number from several approximations. Further, we reported Lorenz numbers calculated from first-principles and experimental results that were termed $L_{Landauer}$. Here, those $L_{Landauer}$ values were used to calculate electronic and lattice contributions to thermal conductivity and the results are shown in Figure S7. A comparison is shown in Figure S7(c) between $L_{Landauer}$ and the SPB-derived Lorenz number calculated using the empirical equation $L_{SPB} = 1.5 + \exp(-|S|/116)$.

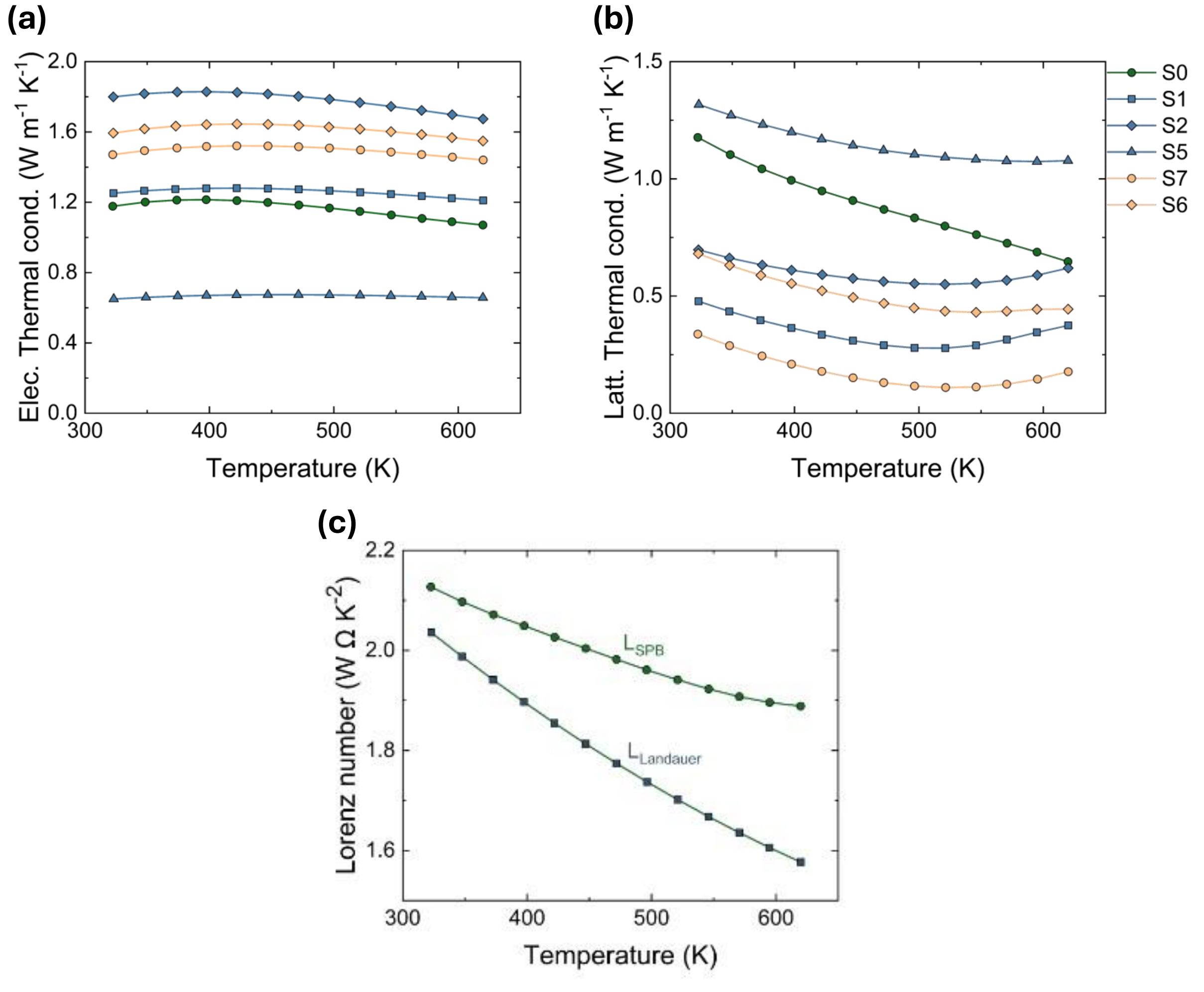

Figure S7: Total thermal conductivity is decoupled into (a) electronic and (b) lattice contributions using the Weidemann-Franz Law. (c) The Lorenz number calculated using the Landauer formalism was used to decouple the electronic and lattice contributions to thermal conductivity.
